# Rapid Acclimation Ability Mediated by Transcriptome Changes in Reef-Building Corals

**DOI:** 10.1093/gbe/evv085

**Published:** 2015-05-15

**Authors:** Rachael A. Bay, Stephen R. Palumbi

**Affiliations:** Department of Biology, Stanford University

**Keywords:** acclimation, transcriptomics, thermal tolerance, coral, climate change

## Abstract

Population response to environmental variation involves adaptation, acclimation, or both. For long-lived organisms, acclimation likely generates a faster response but is only effective if the rates and limits of acclimation match the dynamics of local environmental variation. In coral reef habitats, heat stress from extreme ocean warming can occur over several weeks, resulting in symbiont expulsion and widespread coral death. However, transcriptome regulation during short-term acclimation is not well understood. We examined acclimation during a 11-day experiment in the coral *Acropora nana.* We acclimated colonies to three regimes: ambient temperature (29 °C), increased stable temperature (31 °C), and variable temperature (29–33 °C), mimicking local heat stress conditions. Within 7–11 days, individuals acclimated to increased temperatures had higher tolerance to acute heat stress. Despite physiological changes, no gene expression changes occurred during acclimation before acute heat stress. However, we found strikingly different transcriptional responses to heat stress between acclimation treatments across 893 contigs. Across these contigs, corals acclimated to higher temperatures (31 °C or 29–33 °C) exhibited a muted stress response—the magnitude of expression change before and after heat stress was less than in 29 °C acclimated corals. Our results show that corals have a rapid phase of acclimation that substantially increases their heat resilience within 7 days and that alters their transcriptional response to heat stress. This is in addition to a previously observed longer term response, distinguishable by its shift in baseline expression, under nonstressful conditions. Such rapid acclimation may provide some protection for this species of coral against slow onset of warming ocean temperatures.

## Introduction

Anthropogenic climate change has led to unprecedented shifts in environmental temperature across many ecosystems. One glimmer of hope has come from studies showing that higher resilience to increased temperatures can sometimes be accomplished on the population level through evolutionary adaptation or on the individual level through acclimation (Somero 2010). Acclimation in particular has been studied in the context of rapid climate shifts because it operates within the lifespan of individual organisms, not across generations as in the case of Darwinian adaptation. Yet acclimation itself is under evolutionary pressures, and limits of acclimation are often based on the evolutionary history of the species and population (Meester 1996; Stillman and Somero 2000; Calosi et al. 2010). The role for evolutionary history in acclimation response is consistent with the range of acclimation abilities we see in nature; although the ability to acclimate seems to span every kingdom of life, variation exists both between and within species (Charmantier et al. 2008; Reed and Waples 2010; Somero 2010; Tepolt and Somero 2014).

The value of acclimatory shifts to mitigate climate change impacts, however, needs careful study to not overestimate the potential impact. Specifically, understanding the rate of acclimation relative to the rate of environmental change is crucial. Yet fine scale studies of the acclimation process over short periods of time remain rare. As a result, the link between the nearly ubiquitous ability to acclimate and future climate fitness is incomplete. Acclimation capacity varies greatly among species in ways that may determine susceptibility to future climate change (Somero 2010, 2012). For example, tropical *Petrolisthes* crabs have less acclimatory capacity than their more temperate congeners, making the warm-adapted species paradoxically more susceptible to increases in temperature (Stillman and Somero 2000; Stillman 2003). This flexibility of response to environmental stimuli can take a number of forms, including use of stable protein isoforms, increased production of chaperone proteins, and increased membrane rigidity (Hofmann and Todgham 2010). Differences in acclimation ability are reflected in gene expression responses, where species capable of acclimation show transcriptional plasticity not present in nonacclimating species (Somero 2012).

Different mechanisms underscore different patterns of rate and reversibility of acclimation. In plants, a form of acclimation called “stress memory,” in which a previously stressed individual is less susceptible to future stress, can be categorized into two broad mechanistic groups (Bruce et al. 2007). The first group involves shifting concentrations of proteins or transcription factors that remain at altered levels after an initial stress—a baseline change in expression. In *Arabidopsis*, for example, *CBF* transcription factors induced at low nonfreezing temperatures control freezing tolerance (Thomashow et al. 2001; Cook et al. 2004). The second category consists of changes in chromatin structure such as methylation, histone modification, and chromatin remodeling—processes which might alter the transcriptional availability of a region. For example, histone acetylation in submerged rice plants leads to the upregulation of well-known stress responsive genes (Tsuji 2006). The variance in cellular mechanisms likely contributes to the variance in rates of induction and reversal of acclimation observed in natural systems (Palumbi 1984; Donelson et al. 2011). Acclimation resulting from chromatin modification, for example, can even be passed on to future generations (Boyko et al. 2010; Donelson et al. 2011).

Sessile organisms like plants require especially well-tuned systems for dealing with environmental change. In marine environments, reef-building corals are both sessile and exceptionally long lived, so they will likely experience very different temperatures as climate change proceeds. As foundation species, reef-building corals are essential components of tropical marine ecosystems but live within 1–2 °C of their upper temperature limit (Coles et al. 1976; Jokiel 2004). Climate change has been implicated in the increasing number and severity of coral bleaching events in recent decades. Because ocean temperatures are both steadily increasing and experiencing more temperature anomalies, corals must respond to changes in their thermal environment on both very short and very long time scales.

Both laboratory-based and observational studies lend support for acclimation as an effective mechanism for increasing thermal tolerance in corals. In natural settings, corals subjected to increased temperatures during low tides exhibit higher thermal tolerance than conspecifics in more stable thermal environments (Castillo and Helmuth 2005; Oliver and Palumbi 2011). Controlled acclimation experiments show that individuals subjected to slightly increased temperatures bleach less when exposed to a severe heat stress (Middlebrook et al. 2008; Bellantuono, Hoegh-Guldberg, et al. 2012). Although these studies suggest that acclimation does occur in corals, we know little about the mechanism. Only one study has investigated rapid acclimation in corals at a transcriptional level (Bellantuono, Granados-Cifuentes, et al. 2012). This study examined expression during a chronic stress in individuals acclimated at different temperatures, finding little change in transcription. Despite a substantial shift in heat tolerance, the coral *Acropora millepora* showed only nine genes that were differentially expressed between acclimation treatments. Acclimatization during a reciprocal transplant in *Acropora hyacinthus*, however, did result in a shift in baseline expression; 74 genes were differentially expressed after one year in a new environment (Palumbi et al. 2014).

We conducted a temporal characterization of acclimation in the reef-building coral *Acropora nana.* Recent studies show that corals acclimated to field conditions can increase their thermal tolerance, but do these mechanisms effectively protect corals during rapid bleaching events? Our approach differs from previous laboratory experiments by using a severe, acute heat stress to assay thermal tolerance and transcriptional response. We report that shifts in physiological condition, heat tolerance, symbiont levels, and transcriptome response of the coral *A. nana* occur within 7 days of acclimation, producing a striking increase in heat tolerance. We uncover widespread gene expression shifts associated with acclimation after an acute heat stress. Unexpectedly, physiological acclimation is associated with reduced transcriptome response after heat stress, but no baseline changes in unstressed samples as were found in previous studies. Combining our results with previous studies, we suggest a multistage process for acclimation that can produce a rapid response in the acute heat stress mechanism and a slower response that alters background gene expression and physiological function. Both may slow the impact of ocean warming on corals for a short period of time.

## Materials and Methods

### Acclimation

For acclimation, we used six 81-l flow-through tanks, two replicate tanks for each of three acclimation temperature regimes (supplementary fig. S1, Supplementary Material online). Temperature in each tank was controlled by pumping water from the tank through coils in hot and cold water baths, so that no water was exchanged between tanks. For a control, tanks were held at 29 °C, approximately ambient temperature on the reef at this location (supplementary fig. S1, Supplementary Material online). The second treatment, the “stable” treatment, was held at an increased temperature of 31 °C. The final acclimation treatment, the “variable” treatment, fluctuated throughout the course of the day, mimicking tidal fluctuations and ranging from 29 to 33 °C. We used HOBO pendant temperature data loggers (Onset) to record the temperature of each tank every minute for the duration of the experiment. All tanks were covered in 75% shade cloth to avoid light stress.

**Fig. 1.—:**
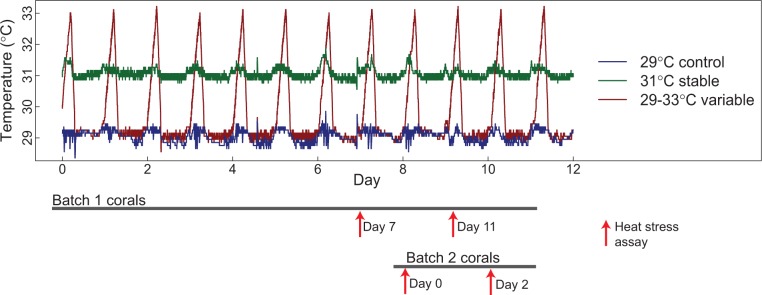
Representative temperature record from acclimation tanks over the course of the experiment. Measurements were taken every minute with HOBO pendant temperature loggers. Gray bars at the bottom show the timing and duration of acclimation for each of two batches of 18 (six per acclimation treatment) *Acropora nana* colonies. Red arrows indicate heat stress assays, labeled by the duration of acclimation for assayed corals.

We collected 18 small colonies (∼15 cm in diameter) of the species *A. nana* from the reef crest on Ofu Island, American Samoa. We evenly distributed the colonies among the six acclimation tanks, resulting in three colonies per tank—six per acclimation treatment. These colonies were assayed for thermal tolerance using an acute heat stress assay (described below) after 7 and 11 days of acclimation. Because of a series of unexpected power outages affecting heat stress tanks, we were unable to assay these colonies during earlier time points. We, therefore, collected a second batch of 18 colonies 1 week later (fig. 1), which we placed in the acclimation tanks and assayed for thermal tolerance after 0 and 2 days of acclimation. In total, this provided us with six heat-stressed and six nonstressed samples for each of three acclimation treatments (29 °C, 31 °C, and 29–33 °C) at each of four acclimation durations (days 0, 2, 7, and 11)—a total of 144 samples.

### Thermal Tolerance Assay

To assay thermal tolerance, two branches were taken from each colony at each time point. One branch was exposed to an acute heat stress—a temperature ramp from 29 to 34 °C over the course of 3 h, followed by 5 h incubated at 34 °C, then a return to 29 °C and incubation at that temperature overnight. The second branch was used as a control—held stable at 29 °C for the duration of the stress experiment. Each heat ramp started at 9:00 AM. No corals died during the acute heat stress, but they experienced varying degrees of bleaching. Samples were collected and preserved at 6:00 AM the following morning. At this time, a fragment from the base of each branch was placed in an RNA stabilizing solution for transcriptomic analysis and the remainder of the branch was preserved in 95% ethanol for chlorophyll a analysis, a proxy for bleaching.

### Chlorophyll a Analysis to Measure Bleaching Response

Fragments of all nonstressed and stressed branches were preserved and transported in 3 ml 95% ethanol and kept in the dark until analyzed. We measured chlorophyll a concentration by measuring absorbance at four wavelengths (632, 649, 655, and 696) with a spectrophotometer (Ritchie 2008). The surface area of each branch was measured by dipping the branch in melted wax twice and weighing the branches after each dip (Stimson and Kinzie 1991). The difference between the two weights is proportional to the surface area. Absolute surface area was determined by comparing these values to a set of standards of known surface area analyzed simultaneously with the branches. Surface areas for each branch were used to normalize chlorophyll a concentrations. We used analysis of variance (ANOVA) to analyze impacts of both acclimation treatment and duration on chlorophyll a concentrations in nonstressed and stressed samples.

### Transcriptome Sequencing and Assembly

A fragment of each sample was preserved for transcriptomic analysis in an RNA stabilizing solution. These samples were stored at 4 °C for 24 h, then transferred to −20 °C for the remainder our time in the field, approximately 1–2 weeks. Samples were transported to Stanford, where they were stored at −80 °C until extraction. We extracted total RNA from each sample using TRIzol reagent (Life Technologies) and created cDNA libraries using the Illumina TruSeq mRNA Sample Prep kit. All 144 samples were barcoded using Illumina adapters and were randomized and pooled into 12 lanes sequenced on an Illumina HiSeq 2000. For de novo assembly, one lane was sequenced as 101-bp paired-end reads, while the remaining 11 lanes were sequenced as 50-bp single-end reads.

Quality filtering, assembly, and mapping of reads followed the pipeline in de Wit et al. (2012). Low-quality (*Q* < 20) portions of reads, as well as short reads (<20 bp) and adapter sequences were trimmed using the fastx-toolkit (http://hannonlab.cshl.edu/fastx_toolkit/index.html). Only paired-end samples were used for de novo assembly using CLC Genomics Workbench (mismatch cost = 1, insertion/deletion cost = 2, length fraction = 0.5, minimum contig length = 200). Noncoral contigs were removed from the assembly using a custom BLAST-based pipeline and the remaining contigs were annotated based on the top BLAST hit to the nr and Uniprot databases (see supplementary materials, Supplementary Material online, for details on filtering and annotation of contigs). Forward reads from the paired-end lane were trimmed to 50 bp, so they could be used along side single-end reads for gene expression. Single-end and trimmed paired-end reads were mapped to the assembly using BWA (Li and Durbin 2009). The number of reads that mapped to each contig with a minimum quality score (*Q* > 20) were counted using an in-house python script. Only contigs with a mean of greater than 5 reads across all samples and a standard deviation less than the mean read count were used in downstream analysis.

### Gene Expression Analysis

We analyzed gene expression separately for heat-stressed and nonstressed conditions. Raw counts were normalized using the DESeq package in R (Anders and Huber 2010). For a broad scale examination of variation, we performed a principle components analysis of all samples. For heat-stressed samples, this revealed nine obvious outliers (three from each acclimation treatment), which corresponded to a single experimental acute heat stress tank run on September 16, 2012, after 2 days of acclimation (supplementary fig. S2, Supplementary Material online). Because this is likely an experimental artifact, we removed these nine samples from downstream analysis. Because samples were evenly divided across heat stress tanks, removal of these samples did not result in loss of any treatment but a halving of sample size for each acclimation treatment at that time point.

To identify genes whose expression was associated with acclimation treatment, normalized counts were used in a permutational ANOVA for each contig, using acclimation treatment and day as factors. Because we are interested in genes with significant differences between acclimation treatments, we examined only contigs in which the acclimation term was significant (false discovery rate [FDR] corrected *P* ≤ 0.01). For this set of genes, we used hierarchical clustering to find groups of co-expressed genes (Spearman correlation > 0.6). The variation in each cluster of co-expressed genes is represented by the centered first principal component of that cluster (Alter et al. 2000). For each cluster, we used ANOVA to test the effects of acclimation treatment and number of acclimation days on gene expression. We also tested for functional enrichment of clusters using GoEast (Zheng and Wang 2008) with FDR < 0.05 to identify overrepresented Gene Ontology (GO) terms for contigs within each cluster.

In addition to analyzing gene expression clusters, we directly tested for differentially expressed contigs between stable (31 °C) and variable (29–33 °C) acclimation treatments. For this analysis, we used only data from days 7 and 11, when acclimation-based differences were most likely to occur. We used the DESeq package to conduct a negative binomial test across all contigs with average coverage of greater than five reads and standard deviation less than the mean.

To examine the heat stress response of acclimation-regulated gene clusters, we grouped all samples and measured the change in expression between stressed and nonstressed samples. We used the DESeq package for normalization, then calculated the heat stress response, defined as log_2_ of the mean difference between heat-stressed and nonstressed expression, for each gene in a co-expressed cluster. We then compared the distribution of heat stress response for a given cluster to a null distribution created from 1,000 randomly sampled groups of the same size as the cluster.

## Results

### Shifts in Bleaching Response After Acute Heat Stress across Acclimation Treatments

**Fig. 2.—:**
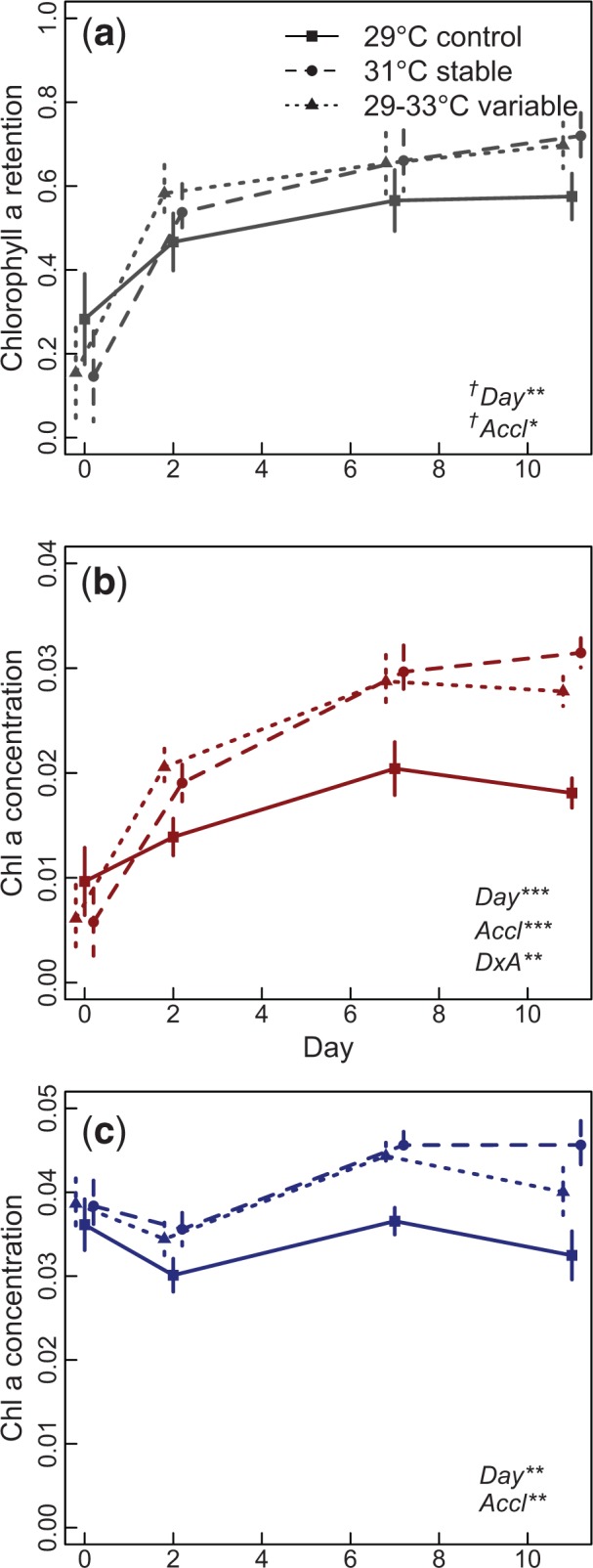
Bleaching resistance in *Acropora nana* branches acclimated under control (29°C), stable (31°C), and variable (29–33°C) conditions. Measurements were taken after 0, 2, 7, and 11 days. Bleaching is measured as the fraction of chlorophyll a retained in a particular colony after heat stress (*a*), calculated by dividing the concentration of chlorophyll a in heat-stressed branches (*b*) by that in nonstressed branches (*c*). Significant factors from ANOVA, “Day,” “Accl”—acclimation treatment, and “DxA”—interaction between Day and Acclimation, are shown in bottom left corner (†Day 0 was omitted for significance testing). Error bars represent standard error across six branches.

Corals acclimated to 31 °C or variable (29–33 °C) temperature regimes showed higher thermal tolerance, measured by the proportion of chlorophyll a retained after heat stress, than corals in the control acclimation treatment (fig. 2*a*). Corals from all three acclimation temperatures show increased heat resistance between days 0 and 2. This is likely due to recovery from either transplant stress or some other nontemperature effect associated with moving colonies from the reef to the tanks. If we exclude day 0 and thus the artifact of transplantation, we observe a significant difference between acclimation treatments in the proportion of chlorophyll a retained in heat-stressed branches compared with nonstressed branches (*P* < 0.05). This pattern is also apparent in the concentration of chlorophyll a from heat-stressed branches alone; branches from 31 °C and variable acclimation treatments had more chlorophyll a after heat stress than the control (*P* < 0.01; fig. 2*b*).

Baseline levels of chlorophyll a also increase in corals acclimated to 31 °C and variable (29–33 °C) acclimation treatments. Analyzing only nonstressed branches, we can examine shifts in chlorophyll a strictly due to acclimation. We find that branches acclimated to increased temperatures have higher chlorophyll a concentration than those acclimated to 29 °C (*P* < 0.01; fig. 2*c*).

### Assembly of De Novo Transcriptome

To analyze transcriptome response to acclimation, we first assembled a de novo transcriptome for *A. nana.* Of 275,243 total contigs assembled, 62,287 were classified as coral contigs based on BLASTN hits to known coral databases, including predicted proteins from the *Acropora digitifera* genome (Shinzato et al. 2011), and excluding contigs that also had significant BLASTN hits to either known *Symbiodinium* databases or the SILVA ribosomal RNA database (see supplementary materials, Supplementary Material online, for detailed methods). Overlapping contigs were joined with an in-house python script, resulting in a total of 59,469 contigs. On the basis of BLAST searches to nr, Uniprot, and Tremble databases, we were able to annotate 20,848 contigs (35%). Table 1 lists statistics for the entire assembly and the final coral-only assembly used in downstream analysis.

**Table 1:** Statistics from De Novo Assembly of *Acropora nana* Transcriptome

Assembly	No. Contigs	Total Length (bp)	N50 (bp)	N90 (bp)
Full	275,243	155,477,256	699	281
Coral	59,479	43,023,267	1,056	318

Note.—“Full assembly” represents values from de novo assembly of all reads from one 101 bp paired-end Illumina HiSeq lane. Statistics for final assembly including only contigs that BLAST to known *Acropora* databases is presented. Overlapping contigs in the coral assembly have been joined using a custom python script.

### Comparison of Gene Expression in Non-Stressed Samples across Acclimation Treatments

To examine transcriptional changes during acclimation, we analyzed the gene expression of nonstressed samples. Of 59,479 total contigs, 28,053 had a mean of greater than five counts and a standard deviation less than the mean across all samples and therefore were used for gene expression analysis. Permutational ANOVA of nonstressed samples yielded no contigs with significant differences across the three acclimation treatments.

### Comparison of Gene Expression in Heat-Stressed Samples across Acclimation Treatments

**Fig. 3.—:**
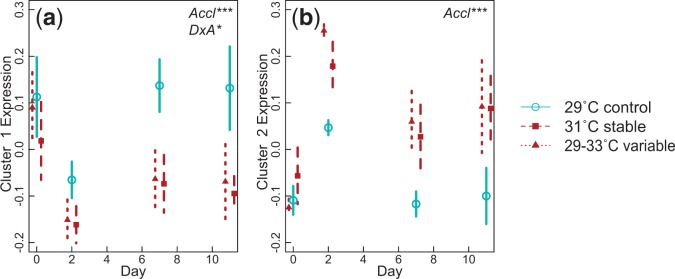
Expression of gene clusters or “eigengenes” with correlated gene expression (>0.6 correlation) in *Acropora nana* samples after acute heat stress. Eigengene expression is represented by the first principle component of the cluster. We grouped this value by acclimation treatment and day to visualize changes in expression of gene clusters due to acclimation. Error bars indicate the 95% confidence interval surrounding the mean. Significant factors are shown in upper right corner for each cluster.

Although there were no differences in gene expression in corals exposed to different acclimation treatments before heat stress tests, gene expression was very different after acute heat stress. The permutational ANOVA highlighted 893 contigs showing expression levels after heat stress that differed between acclimation treatments (FDR corrected *P* < 0.01). Of these, 780 contigs could be clustered into two sets that each represented co-expressed genes varying across colonies and acclimation treatments (fig. 3). Co-expression of genes is often a result of shared regulatory mechanisms, for example, common transcription factors. The two clusters of genes in our data show precisely opposite patterns—while cluster 1 has decreased expression in acclimated samples, cluster 2 has increased expression—suggesting the regulatory control mechanism may be the same while the direction of expression change is opposite. We see a spike in expression of both clusters at day 2, which we attribute to stress after being transplanted. In both clusters, all acclimation treatments show similar expression levels at day 0, but by days 7 and 11, stable (31 °C) and variable (29–33 °C) acclimation treatments have diverged from the control acclimation treatment. Cluster 1 contains just 71 contigs, which were not significantly functionally enriched for any GO terms. Cluster 2 contains the majority of clustered contigs (709 contigs) and the GoEast analysis finds significant enrichment for GO terms related to carbohydrate metabolism and ribosomal RNA processing (supplementary table S1, Supplementary Material online).

### Comparison of Bleaching and Gene Expression between Stable and Variable Acclimation Treatments

Although experiencing increased acclimation temperatures changed both bleaching phenotype and gene expression after heat stress, we observed no differences between stable (31 °C) and variable (29–33 °C) acclimation temperatures. Bleaching phenotypes were not significantly different between these acclimation treatments. We also directly compared gene expression between 31 °C and variable conditions at the later days 7 and 11, when acclimatory shifts are most likely to be observed. These tests yielded no genes significantly differentially regulated in either the heat-stressed or nonstressed conditions (FDR adjusted *P* < 0.05).

### Comparison of Gene Expression Before and After Acute Heat Stress

**Fig. 4.—:**
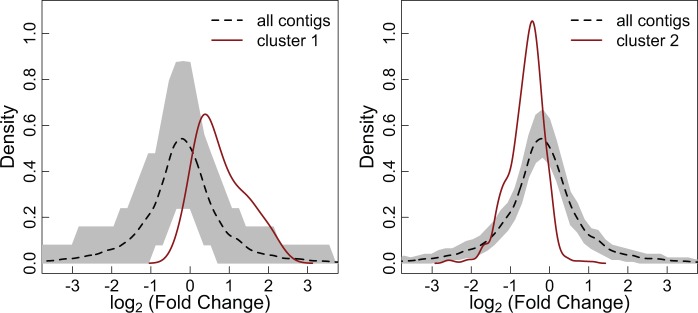
Heat stress response of acclimation-affected gene clusters. The red line shows the probability density of the log_2_ fold change between stressed and nonstressed samples for a given cluster, identified by comparing gene expression of heat-stressed samples across acclimation treatments. The dotted black line shows the null expectation—the log_2_ fold change between stressed and nonstressed samples for all contigs—and the gray envelope shows the 95% confidence interval for a randomly sampled group of contigs of the same size as the cluster. The left panel shows cluster 1 (*n* = 71), which is upregulated during heat stress, whereas the right panel shows cluster 2 (*n* = 709), which is downregulated during heat stress.

**Fig. 5.—:**
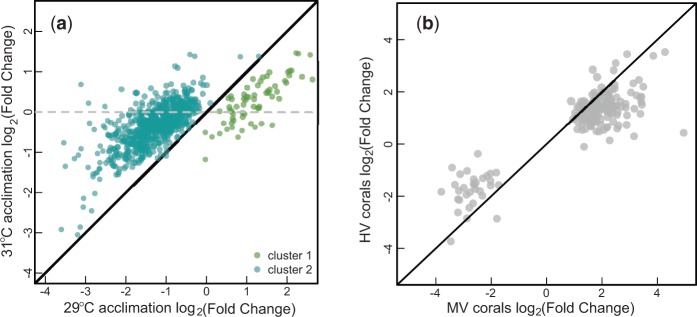
Transcriptional damping as a heat stress response after acclimation. (*a*) On the *x*-axis is log_2_ expression from heat-stressed branches of *Acropora nana* divided by expression from nonstressed branches after 11 days of 29°C acclimation. The *y*-axis shows the same measure for branches acclimated to 31°C for 11 days. Genes in different co-expressed clusters are represented by different colors. A similar pattern is seen under variable acclimation conditions (supplementary fig. S4, Supplementary Material online). (*b*) Plot redrawn from Barshis et al. (2013) showing very similar expression patterns. Here, the *x*-axis represents response of genes to acute heat stress in a population of corals living in a cooler part of the back reef (MV corals). The *y*-axis represents response of the same genes to the same acute heat stress in a population of corals living in a warmer part of the back reef (HV corals).

Heat-stressed and nonstressed branches had dramatically different transcriptional profiles. The result is that the total number of reads that could be mapped to the reference transcriptome was larger for the nonstressed samples (*P* < 0.01; supplementary fig. S3, Supplementary Material online)—transcriptomes generated from nonstressed branches had an average of 3.02 million reads mapped compared with 2.26 million for stressed samples. Of 28,053 contigs, 22,338 (79.6%) yielded significant results (FDR = 0.05) in a negative binomial test for differential expression during heat stress. The difference in total read count is likely the result of a drastic change in expression across many genes, altering total transcription levels. Since normalization methods assume that most genes are not differentially expressed, a gene-by-gene analysis of heat stress expression is not reliable in this case. We can, however, compare the magnitude of change during heat stress across groups of genes. We, therefore, examine the response to acute heat stress of genes in the two clusters we identified as differentially expressed across acclimation treatments. These broad-scale comparisons show us that cluster 1 is upregulated after heat stress (*P* < 0.01) compared with a random sample, while cluster 2 is downregulated after heat stress (*P* < 0.01; fig. 4). The stress response seen in the 29 °C acclimated samples is dampened in coral colonies acclimated to higher temperatures (fig. 5; supplementary fig. S4, Supplementary Material online); the magnitude of expression during heat stress is smaller for samples acclimated to either 31 °C or variable (29–33 °C) temperatures. Upon further investigation, we see that this muted response is not the result of a change in baseline expression, but differential expression after heat stress in individuals acclimated to higher temperatures (supplementary fig. S5, Supplementary Material online).

## Discussion

Our results show that exposure to increased sublethal temperatures can rapidly increase tolerance to acute heat stress in the coral *A. nana* and that daily pulses in temperature have the same effect. Changes in heat resistance and chlorophyll a content occur within 7–11 days. Yet, these strong physiological changes occur with little change in baseline gene expression. These results are in parallel with the one other available study on heat acclimation and transcription in corals (Bellantuono, Granados-Cifuentes, et al. 2012) but are in contrast with long-term acclimation studies that show strong shifts in baseline gene expression (Barshis et al. 2013; Palumbi et al. 2014).

However, our additional experiment, in which we examined transcription after acute heat stress, shows a far different pattern. Nearly 900 genes showed effects of acclimation in their expression after exposure to heat stress. Although the difference in species across these studies must be considered—Bellantuono, Granados-Cifuentes, et al. tested *A. millepora*, Palumbi et al. (2014) studied *A. hyacinthus*, and this study used *A. nana*—short-term acclimation does in fact occur in *A. hyacinthus* and other congeners and long-term acclimatory effects occur in *A. nana* (Bay R and Palumbi S, unpublished data) suggesting parallel mechanisms within the *Acropora* lineage. Together, these results suggest two different modes of heat acclimation in corals, a short-term mode that alters gene expression after an acute stress and a long-term mode that alters baseline gene expression before acute stress.

### Stable and Fluctuating Temperatures Increase Thermal Tolerance

We show that thermal tolerance increases quickly—on the scale of 1–2 weeks after exposure to sublethal increases in temperature. In addition, we found as much acclimation in a variable temperature regime (29–33 °C) as in a stable increased temperature (31 °C). Based on field observations, many studies have hypothesized that large magnitude fluctuations in temperature may result in higher thermal tolerance in corals (Castillo and Helmuth 2005; Oliver and Palumbi 2011). Acclimation to fluctuating temperatures has also been shown to alter gene expression in other organisms. For example, Podrabsky and Somero (2004) found that different classes of heat shock protein were upregulated during acclimation to fluctuating and constant temperatures in the killifish *Austrofundulus limnaeus.* However, we find no evidence for such differences within our data; no significant difference in either thermal tolerance or gene expression was observed between stable and variable acclimation treatments.

At a different site on Ofu Island, Palumbi et al. (2014) found increased thermal tolerance in individuals transplanted for over a year to a location that exceeded 31 °C an average of 0.7% of the time compared with a less variable location, which exceeded 31 °C just 0.1% of the time. Using the same metric, our acclimation treatments appear very different from one another: the stable acclimation treatment exceeded 31 °C 41% of the time, while the variable treatment only exceeded 31 °C 18% of the time. Yet no differences in either bleaching or gene expression profiles were observed between these two treatments. This suggests that perhaps thermal tolerance does not increase linearly with either the magnitude of temperature increase or an integrated temperature over time but may rather be a “switch” that occurs when the organism experiences a temperature threshold.

### Stability of Gene Expression in Nonheat-Stressed Branches during Acclimation

Although we see a marked difference in thermal tolerance phenotype after acclimation to increased temperatures, we do not observe a corresponding change in baseline expression in nonstressed samples. Shifts in baseline gene expression during temperature acclimation have been observed in plants, for example, in the induction of genes that convey freezing tolerance after acclimation to decreased temperatures (Thomashow et al. 2001; Cook et al. 2004). Long-term field acclimation experiments in a congener, *A. hyacinthus* have found changes in expression in a broad array of genes after acclimation to increased temperatures (Barshis et al. 2013; Palumbi et al. 2014). Our data do not show this pattern; the ANOVA does not yield a single contig with acclimation-associated expression in nonstressed samples. The mechanism of acclimation in our experiment appears to be different than in previous studies, or the response may be incomplete, perhaps due to the short duration of acclimation.

### Mechanisms of Acclimation

The observation that recent thermal history alters thermal tolerance is consistent with decades of work on a broad range of organisms (Otto 1974; Stillman and Somero 2000; Bruce et al. 2007). In many cases, both stable and variable increased temperatures can increase heat tolerance, often through changes in gene expression, for example, through plasticity of heat shock proteins (Tomanek and Somero 1999; Podrabsky and Somero 2004). Indeed, this type of stress memory has been documented in other species of corals (Middlebrook et al. 2008; Bellantuono, Hoegh-Guldberg, et al. 2012). Bellantuono, Granados-Cifuentes, et al. (2012) found only a small number of genes differentially expressed between preconditioned and nonpreconditioned *A. millepora.* They also found plasticity of gene expression—the magnitude of stress response in nonpreconditioned corals was greater than that of preconditioned corals at a handful of stress responsive genes.

Our findings are consistent with the idea of dampened stress response, with both acclimation-regulated heat stress clusters showing a lesser magnitude response after increased temperature acclimation. In fact, in a number of cases contigs that were stress responsive after 29 °C acclimated conditions responded less to acute heat stress after acclimation to higher temperatures (fig. 5).

The majority of acclimation-associated genes belong to a single cluster containing 709 contigs. Genes in this cluster decreased after heat stress but much less so after acclimation. This cluster is dominated by genes for ribosomal RNA processing and carbohydrate metabolism. A dampened response to acute heat in this case may reflect lower stress levels and higher metabolism in acclimated colonies. Previous studies have shown that rRNA synthesis rate can be altered in response to a change in intracellular energy status, such as during environmental stress (Murayama et al. 2008; Boulon et al. 2010). Ribosomes are responsible for synthesizing new proteins. Perhaps, heat acclimated samples experience less stress-related protein damage and therefore have different energy requirements than samples acclimated under control conditions.

Within this same cluster is the only heat shock protein we find to be associated with acclimation, *HSP75.* As with other genes in this cluster, this contig decreases after heat stress but to a lesser magnitude in acclimated organisms. Other studies have found important roles for multiple heat shock proteins in acclimation of snails (Tomanek and Somero 1999), fish (Podrabsky and Somero 2004; Fangue 2006), plants (Larkindale and Vierling 2007), and many other organisms including corals (Palumbi et al. 2014). Our data, however, find only a single contig with significant expression differences between acclimation treatments. It is possible that the induction of heat shock proteins occurs earlier during thermal stress and we did not see this signal because we sampled the following day. Seneca and Palumbi (2015) found that 19 heat shock contigs in *A. hyacinthus* increased 5 h after acute heat shock, but only three remained at high level after 20 h.

A second cluster of 71 contigs increases after acute heat stress and shows a dampened increase in expression when acute heat stress follows acclimation to high temperature. Because nearly 60% of these contigs had no annotation, we found no significant GO categories for this cluster. However, five contigs in this cluster have known functions in ubiquitination, or protein recycling, including a PHD finger protein, a ubiquitin protein ligase, two RING finger proteins, and a ubiquitin-like protein. Studies in several organisms, including yeast model systems, have shown that expression of ubiquitins can be related to thermal tolerance (Barshis et al. 2010; Shahsavarani et al. 2012).

### Transcriptional Dampening and Two Stages of Thermal Acclimation

Whether populations persist during anthropogenic climate change will depend partly on the relative rates of increase of thermal tolerance and of environmental temperatures. Our results show that substantial physiological acclimation begins quickly, within 7–11 days, and can increase thermal tolerance and alter gene expression patterns. This suggests that corals can track environmental temperatures better than previously believed. Such rapid change in heat sensitivity runs contrary to coral bleaching models based on fixed thermal tolerance that are currently used to predict coral bleaching and climate change response (Liu et al. 2013; Logan et al. 2013). However, the limits to acclimation’s ability to increase thermal tolerance remain unknown and should be examined in future studies.

Thermal tolerance in nature has two components: an adaptive component determined by fixed differences at the individual level such as genotype, development, or epigenetics and an acclimatory component determined by physiological responses to the local environment. Previously, we showed that thermal tolerance in a different coral, *A. hyacinthus* included both acclimation and fixed effects (Palumbi et al. 2014). We identified over 100 genetic variants that were associated with thermal tolerance in populations of this species living in warm back reef pools (Bay and Palumbi 2014) but also that transplanting colonies between pools changed colony response to acute heat stress (Palumbi et al. 2014).

The data we present here show very rapid physiological change but also suggest that the regulatory mechanism of temperature acclimation may have several phases. Our study focuses on rapid responses and finds few baseline changes in gene expression in the first week. In contrast, studies of longer term acclimation for corals in this environment show substantial baseline change in gene expression (Barshis et al. 2013; Palumbi et al. 2014). Despite these differences, both of these acclimatory mechanisms result in a strikingly similar dampened transcriptional response to heat stress in individuals that have adjusted to higher temperatures (fig. 5*A* and *B*). In the case of short term acclimation, transcriptional dampening results from a lower reaction of these genes to acute stress (fig. 5*A*). In the case of longer term acclimation (fig. 5*B*), transcriptional dampening occurs because these genes are already upregulated in acclimated colonies, even before acute heat stress. We termed this latter response “frontloading” (Barshis et al. 2013) and hypothesized that it represented a baseline gene expression strategy for long-term heat acclimation. Our current experiments with *A*.* nana* suggest that frontloading occurs after a longer acclimation period than 11 days, whereas transcriptional damping occurs quickly.

The ultimate response of populations to climate change will depend on the relative roles of adaptation and acclimation. Because adaptation takes much longer, it is unlikely that adaptation alone will keep pace with climate change. Acclimation can shift individual colony responses over a time scale as short as 1 week (see also Bellantuono, Hoegh-Guldberg, et al. 2012), perhaps acting to provide rapid seasonal adjustments in a coral’s heat tolerance or even respond to short-term bleaching temperatures that accompany el Niño events. Our results and those of Bellantuono, Granados-Cifuentes, et al. (2012) suggest that this rapid acclimation involves transcriptional dampening without baseline gene expression changes.

However, acclimation over longer periods includes a set of baseline expression changes that may more significantly restructure a coral’s daily metabolism and growth. Perhaps it is a sequential response that begins with changes in gene expression during heat stress, followed by changes in baseline expression, followed eventually by genetic adaptation, that may allow populations to persist in a changing environment.

## Supplementary Material

Supplementary methods, table S1, and figures S1–S5 are available at *Genome Biology and Evolution* online (http://www.gbe.oxfordjournals.org/).
